# Book Reviews

**Published:** 1862-07

**Authors:** 


### OUR LIBRARY TABLE.

*The A, B, C of Thought: Consciousness the Standard of Truth; or, Peerings into the Logic of the Future.* By the Rev. W. G. Davies. London, Williams and Norgate. 1862. Sm. 8vo. Pp. 144.

This is a charming and most suggestive little book. Placing himself on logic as a standing-point, Mr. Davies has endeavoured to take a comprehensive view of the psychical domain around; and not resting satisfied with merely examining mental processes in their results, he has penetrated from logic wherever he could into psychology. As a consequence he finds that the logic and the psychology have not always harmonized; hence he is led to modify, in several instances, the ordinary doctrines of logical science in such a manner as his psychological researches seemed to suggest. He "cannot conceive," he observes, "though the contrary opinion is held by high authorities, that the laws of thought can be fully determined otherwise than by following the method here observed, that is, tracing every mental process to its source, by a searching and exhaustive analysis."

"Can we from reflection on our own mental phenomena—with the aid, of course, of philosophers in the same field—gain possession of those universal truths which constitute a science of the mind's operations?" This is the important question which Mr. Davies attempts to solve. He divides his work into three parts, the first of which is devoted to an analysis of Consciousness; the second to an examination of Perception and Conception in relation to Form; and the third to Reason and its Forms.

In the prefatory remarks to the second part, Mr. Davies observes :—

"Mr. J. S. Mill declares 'that logic, because it has no concern with the intimate facts of the mind, is common ground, on which the partisans of Hartley and of Reid, of Locke and of Kant, may meet and join hands.' And again he says, 'that logic has no concern with the nature of the act of judging or believing; the consideration of that act, as a phenomenon of mind, belongs to another science.' Bishop Thomson also states that 'our conceptions are formed from single objects; how do we come to know these? The logician replies, that it is not his business to show how.' Mr. Devey also declares that all these questions 'involve metaphysical theories, concerning which the greatest minds have differed, and are quite extraneous to logic, which deals with principles about whose cogency there cannot be the slightest doubt.' Now we have made it our duty to attempt precisely the very task which these logicians assign to the metaphysician; that is, to interpret logic by the light of psychology. And if there be anything worthy of acceptance in these contributions, it arises chiefly from a steadfast adherence to the method which we have deemed it right to pursue." (p. 43.)

We shall not attempt to follow Mr. Davies in detail, but content ourselves with citing one or two examples of his method of dealing with the great subjects which come within the scope of his work. These better serve to indicate its character than any meagre description or criticism. Writing of the order in which Knowledge grows, Mr. Davies says :—

"Although, as we have shown above, a certain era is marked by the activity of this faculty rather than of that, it must not be forgotten that all the faculties manifest, in regard to undecided points, a spontaneous, or transitional energy, which greatly influences the most advanced thinking of that era. The spontaneous and transitional thoughts of a period, whether in religion, politics, or science, serve as so many checks against presumed finality and narrowness of view. The grandest truths are the last to enter their final stage; whereas truths inferior enter that stage at a comparatively early period. Now some persons pronounce that discovery terminates with truths of the latter kind. A sensational philosopher is too ready to do this; and to boast of his accepting nothing till it shall be proved. But when positive knowledge is so scant, by so doing, he throws a thick mist of negation upon all that makes man's life full and joyous. The antidote to this narrow philosophy—a philosophy which endeavours to impose upon mankind a gaunt skeleton of truth, in room of the lovely Divinity herself—is *that portion of a nation's thoughts which has not yet entered upon its final stage; and which, by loudly asserting its superiority, deters inquirers from limiting the domain of knowledge, as in vision, space is limited by the limitation of the sense of sight.* 'Thousand to one (says Lessing) the goal of your philosophy will be the spot where you become weary of thinking any further—a remark which should caution us not to be too hasty in interdicting any branch of investigation as transcending our faculties, and not to fix the boundaries of demonstrative knowledge without *very* sufficient grounds.' Our better nature will inevitably—

"Urge us on,
With unremitting labour to pursue
Those sacred stores that wait the *ripening* soul,
In Truth's exhaustless bosom." (p. 128.)

Again, on the Veracity of Consciousness Mr. Davies remarks :—

"It is surprising to find how frequently philosophers have been within sight of new land, and yet have failed to see it. It seems strange, for example, that to a mind like Sir Wm. Hamilton's, the thought did not occur, that 'the reasoning,' on which was based the assurance that the actual manifestations of consciousness could not be doubted, admitted of being developed into a reasoning which proves, what Reid and Stewart felt to be fact, that the existence of the external world is not less certain than our own existence; which proves that the *testimony* of the witness, consciousness, is, to all intents, as correct, as the *existence* of the witness is beyond doubt. For, let us ask, what is the ground of the conviction, that a fact of consciousness cannot be doubted? A deliverance of consciousness, namely, a reasoning. Is this deliverance trustworthy? Reason, reflectively operating, waits for an answer; and Reason asks this question, because it craves for its appropriate evidence. Till this evidence shall be supplied, even Sir Wm. Hamilton's argument is not unassailable. It is only an answer to direct doubt. It leaves reflective doubt still in possession of the field. It allows the sceptic still to ask, do my faculties deceive me or do they not? Can a satisfactory answer be afforded to this question? Let us see.

If we question the veracity of a deliverance of consciousness, it behoves us to *ascertain by Induction* which is logically prior to the other, the veracity or the deliverance. We then discover that the veracity is presupposed by the deliverance. Veracity is inseparable from consciousness when the latter is possessed of direct or transitive assurance. No other attribute of consciousness underlies its veracity. That is primary. Would we *prove* this veracity? The attempted proof presupposes the veracity it would prove. Would we *disprove* this veracity? The attempted disproof presupposes the veracity it would disprove. The foundation of all knowledge, knowledge of *self*, or knowledge of *not-self*, the primary principle presupposed alike by consciousness when revealing its own existence, or that of its objects, be the latter mental, organic, or extra-organic, of the man, or out of the man is—VERACITY. Consciousness, therefore, is indeed The Standard of Truth. May we hope that we have succeeded in some degree in pointing out how it possesses this momentous function; and that our labour will not be entirely in vain?"

Finally, in concluding, Mr. Davies says of the objects of his work:—

"These contributions have a tendency to mediate between the rival schools of philosophy, and thus to produce unanimity where now there is conflict, and by this unanimity to further the advancement of the higher truths. They have therefore a tendency to rebuke one-sidedness, and the elevation of half-truths into whole truths. They seek to curb the extravagances of *private* judgment by an appeal to the universal judgment of man, to consciousness as the Truth-Standard. And by supplying such a standard, they enable us to detect all departures from it whether in the sane or the insane mind. They award to sensational truths the honour of being in a more advanced stage of development than rational truths, those apprehended by Reason. But they claim for these last a decided superiority in rank, and account for their less forward growth by the law, that the simple and general is prior in scientific development to the comprehensive and superior. They uphold the full authority of the dominant, yet vindicate the claims of the subservient to all due consideration, that is, to immunity from that hurtful ascendency, hurtful even to itself, which the dominant has too frequently exercised over that which is absolutely necessary to the very existence of the dominant."

These great objects have been most thoughtfully and conscientiously carried out by Mr. Davies; his book is eminently *sound-minded*. Portions of the work originally appeared in this Journal, and the "Journal of Mental Science."

*Clinical Essays.* By BENJAMIN W. RICHARDSON, M.A., M.D., Senior Physician to the Infirmary for Diseases of the Chest. (*Asclepiad*, Vol. I.) 8vo. London, 1862. pp. 272.

This volume is the first of a series, containing essays on subjects relating to medical science in its various departments, which Dr. Richardson proposes to publish from time to time. It will be his object, he states, always to consult the requirements of practical medicine; yet, at the same time, endeavour to weave into the subjects discussed, such matters bearing on the current theories of medicine as shall tend to open the way to new and more comprehensive views, and to a sounder and more rational practice.

In the present volume the Essays are seven in number, and are devoted to the following subjects: (1) Subclavian Murmur; (2) on a Diseased Condition of the Nails; (3) on Reduplication of the Second Sound of the Heart; (4) Contributions towards a more perfect Clinical History of Scarlet Fever; (5) on Pulsatile Pulmonic Crepitation; (6) on Uræmic Coma; and (7) on Cardiac Apnœa. The whole of these Essays are characterized by that clearness of thought, precision yet vigour of expression, thorough practicalness and yet large originality which so eminently distinguish the writings of the author. The volume is, indeed, to be commended in an especial manner to men in the active practice of their profession, to whom these Essays, and more particularly the three on Scarlet Fever, Uræmic Coma, and Cardiac Apnœa, will prove of unusual interest and value. It would be unjust to Dr. Richardson, still more unjust to the Essays, to make any attempt to analyze the contents of this volume. They must be read in detail to be rightly appreciated. One illustration, however, we may give in confirmation of our remarks on their practical bearing. It refers to the treatment of Scarlet Fever.

After pointing out that the essence of the treatment of this disease in its early stages is to produce profuse excretion by the skin; "in other words, to expedite as quickly as possible the expulsion of the products of a malchemistry from the blood," and indicating the readiest modes of effecting this, Dr. Richardson says:—

"But we must never forget that there is another element in the disorder, which may prove the fatal element; I mean the deposition of fibrine in the right cavities of the heart.

"To meet this tendency to deposition, to prevent its occurrence, two remedies seem to me to be most reliable. The one is ammonia, as first recommended by Dr. Peart, and more recently by Dr. Urtt; the other is acetic acid, as recommended by Mr. Isaac Baker Brown. I have often put these remedies into use, in practice, and I am bound to say that they are both most effectual. It would be difficult to decide which is the best.

"The action of these remedies at first sight may seem contradictory, but it is not so. Both acetic acid and ammonia have one property in common, that of holding the fibrine of the blood in solution. Both medicines also tend to eliminate carbonic acid; for ammonia, when freely administered, escapes from the skin, the breath, and perhaps the urine, in the form of carbonate; and acetic acid is decomposed entirely, escaping from the urine as carbonic acid in combination with a base. Further, by keeping the blood fluid, they favour elimination from every excreting surface. I have used with equally good effects a combination of these two remedies with an excess of ammonia, as in doses of two fluid-drachms of the liquor ammoniæ acetatis, with from three to five drops of liquor ammonia, in a liberal quantity of distilled water.

"It is of importance in these exhibitions to administer the agent in small and frequently repeated doses, so that the blood may always contain the remedy; for if the doses be given with intervals of three or four hours apart, the decomposition and elimination of the substance is over so quickly, that the system is left free of the effects during the greater part of the period between each dose. In a word, the secret of administration consists in putting aside the idea of a medicinal dose altogether, and in offering the remedy as a pleasant drink, rather than as a nauseous draught.

"If the ammonia treatment be selected, there are two points particularly to be observed. The doses must be followed up until the agent is very distinctly presented in the breath. This presence may be determined by the holding of a glass rod moistened with hydrochloric acid for the patient to exhale on; when, if much ammonia be expired, the white characteristic fumes will be developed. This test, however, is not very satisfactory. It is better to place a drop of pure hydrochloric acid on a microscope slide, and allow the patient to exhale some twenty or thirty times over the acid surface. Then, the glass slide being gently dried before the fire, or near a spirit-lamp, there will be left a crystalline deposit, which, on microscopical examination, will present the characters depicted in plate II., figure 1.

"To effect this examination of the breath, Mr. Toogood, of Mount Street, has constructed under my direction, a very simple pocket-tube, delineated in figure 2, of plate II.

"The tube is in fact a strong test-tube, with a neck holding a stopper perforated in two places. One of the openings is armed with a mouthpiece, and the whole is covered in with a well-fitted glass cap. When the tube is prepared for use, a microscope slide is moistened at one or two points with a minim of pure hydrochloric acid, and is inserted in the tube; the stopper and glass cap are then adjusted, and the whole may be carried in the pocket safely. When it is to be applied, the glass cap is removed, and the patient is made to expire gently by the mouth through the mouthpiece some twenty or thirty times. The cap may then be replaced, and the tube removed altogether, and examined at once, or retained for a time. Before microscopical examination the cap is removed, and the tube, the stopper still in it, is placed near a fire, or at a little distance from the flame of a spirit-lamp, in order to allow the chloride that may be formed to crystallize on the slide. When this is effected, the stopper is removed, and the slide is ready to be placed under the microscope for examination. The advantages of this tube are:—first, that it is portable; second, that in use nothing but the expired air of the patient comes into contact with the acid; and third, that the evaporation and crystallization are performed with as little exposure to the air as is possible.

"A few experiments with this tube on healthy persons will indicate that under most circumstances crystals of chloride of ammonium are produced after expiration through the tube when the slide is charged with hydrochloric acid. But practice soon points out the extreme amount of the crystalline body that can be produced in health by a fixed number of expirations; so that comparisons between this production, and that occurring after the administration of ammonia, are easily and practically learned. In health, the crystalline deposit is thin and scattered; after free administration of ammonia, if the action of the skin or kidneys, or of both, be not profuse, the slide is frosted with the salt.

"In administering ammonia it is essential that the medicine be pushed to the extent of showing a free evolution in the breath. But while this point is to be insisted on, it is essential that there be a limit to the administration; for if it be carried to an extreme, two evils are induced. In the first place, the ammonia by its presence stops, to a considerable extent, the process of oxygenation; and, secondly, it acts by its solvent power on the blood-corpuscles. It is well therefore, nay requisite, also to examine the blood microscopically during the administration; when, if the red corpuscles while still in motion, present the appearances marked A, B, C, D, E, F, G, in figure 3, plate ii.—that is to say, if the discs be crenate, or irregular, or transparent, or collapsed, or oval, or very diffuse—the indications are given that the alkali is becoming dangerous, and requires to be suspended." (pp. 104—8.)

*Of Anagrams, a Monograph Treating of their History from the Earliest Ages to the Present Time; with an Introduction, containing numerous specimens of Macaronic Poetry, Punning Mottoes, Rhopalic, Shaped, Equivocal, Lyon, and Echo verses, Alliteration, Acrostics, Lipograms, Palindromes, Bouts Rimés.* By H. B. WHEATLEY. Williams and Norgate. 12mo., pp. 186. 1862.

This is one of the daintiest little books, brimful of the quaintest conceits. It was a happy idea of Mr. Wheatley to form a collection of these curious literary follies, and to serve them up in so appetizing a form. Insane follies must at all times be gauged by sane follies; and here we have many of the most curious illustrations of the latter. Anagrams, moreover, have a remote place among the determining causes of insanity. Thus Addison relates, in his papers on "False Wit" (*Spectator*, Nos. 58, 63), the unhappy case of an unfortunate lover who expended much time over an anagram of his mistress' name; but when he had completed the task, and presented the result to her, he was shocked to find that he had spelt her name incorrectly, its real orthography being "Mary Bohun," while he, no doubt much to the fair one's annoyance, had framed his elaborate transposition from the prosaic letters of "Moll Boon." "The lover," says Addison, "was thunderstruck with his misfortune, insomuch that in a little time after he lost his senses, which indeed had been very much impaired by that continued application he had given to his anagram."

But anagrams and allied literary follies possess a mysterious philosophy which is not to be rashly slighted. "Plato," writes Disraeli in his 'Curiosities of Literature,' "had strange notions of the influence of anagrams when drawn out of persons' names, and the later Platonists are full of the mysteries of the anagrammatic virtues of names. The chimerical associations of the character and qualities of a man with his name anagrammatized, may often have instigated to the choice of a vocation or otherwise affected his imagination."

An illustrious confirmation of the Neo-Platonic notions is to be found in the anagram of FLORENCE NIGHTINGALE, which reads *Flit on, cheering angel.*

Of the serious uses to which anagrams and word-playing have been applied, we may quote the following illustrations:—

"Sir Thomas Herbert, in his 'Travels in Africa and Asia,' (folio, London, 1677,) speaking of the derivation of the word Ophir, says—'Yea, to strengthen that imagination, others suppose that by the word *Sophyra* (which is Ophyr anagrammatized), mentioned in the lxxii. interpreters, is intended or meant *Soffala* or *Sophura*, as to attain their ends they wrest it; albeit, St. Jerome by that name intends *Sepher*.'" (p. 73.)

Again—

"A passage of Scripture arranged chronogrammatically was made the vehicle for a prophecy by Michel Stifelius, a Lutheran minister, at Wurtemburg, who foretold that, on the 3rd of October, 1533, at ten o'clock in the morning, the world would come to an end. The passage from which he elicited this wonderful, and, as it proved, inaccurate prediction, is in John xix. 37.—'They shall look on him whom they pierced.'—(VIDebVnt In qVeM transf IXer Vnt, making MDXVVVVIII., or 1533); but the month, the day, and the hour, seem only to have existed in the excited imagination of the worthy Stifelius himself. There is a rider to this anecdote which may be thus related:—On the day that Stifelius predicted the end of the world, a very violent storm arose while he was preaching to the congregation, who believed his prophecy was coming to pass, when, lo! suddenly the clouds disappeared, the sky became clear, and all was calm except the people, whose indignation was aroused, and they dragged the prophet from his pulpit and beat him sorely for thus disappointing them.'" (p. 5.)

Here is a specimen of alliterative versification directed at the profession.

"Medical men, my mood mistaking,
Most mawkish monstrous messes making,
Molest me much; most manfully
My mind might meet my malady:
Medicine's mere mockery murders me."

Such are a few samples of the contents of Mr. Wheatley's pleasant little volume. These jottings of literary leisure are very piquant things, and Mr. Wheatley has broken good ground, which we trust he will not suffer to lie needlessly fallow.

*Erasmus Darwin, Philosopher, Poet, and Physician.* A Lecture to the Literary and Philosophical Society of Whitby. By John Dowson, A.M., M.D. London: H. K. Lewis. pp. 181.—A very useful sketch of a notable character. Dr. Dowson's Lecture is, we are glad to say, but the forerunner of a fuller account of the life and works of Darwin.

